# Antimicrobial resistant bacteria in wild mammals and birds: a coincidence or cause for concern?

**DOI:** 10.1186/2046-0481-67-8

**Published:** 2014-04-25

**Authors:** Shaun Smith, Juan Wang, Séamus Fanning, Barry J McMahon

**Affiliations:** 1 UCD School of Agriculture & Food Science, University College Dublin, Belfield, Dublin 4, Ireland; 2 UCD Centre for Food Safety, UCD School of Public Health, Physiotherapy & Population Science, University College Dublin, Belfield, Dublin 4, Ireland

**Keywords:** Herring gull, *Larus argentatus*, Deer, Antimicrobial resistance, *Escherichia coli*

## Abstract

**Background:**

The emergence and dissemination of antimicrobial resistance (AMR) is a growing concern to public and animal health. The contribution attributable to wildlife remains unclear. In this study two unrelated wildlife species herring gulls (*Larus argentatus*) and a hybrid deer (*Cervus elaphus x Cervus nippon*) were investigated for the presence of *Escherichia coli* expressing an AMR phenotype.

**Findings:**

Bacterial isolates resistant to β-lactam compounds were identified in both animal species and the production of functional β-lactamase was confirmed using nitrocefin. The prevalence of resistant isolates was higher in herring gulls (87%) compared to deer (31%). Resistance to this class of antibiotic was found only in non-pathogenic *E. coli* in herring gulls and in both pathogenic and non-pathogenic *E. coli* strains in deer.

**Conclusions:**

The presence of AMR in wildlife has implications for public health, food safety and potable water source protection among others.

## Findings

Antimicrobial resistant (AMR) bacteria are a growing problem worldwide for public health, industry and the environment [1]. Emergence of species such as *Escherichia coli* that are resistant to extended-spectrum βeta-lactams and fluoroquinolones is cause for serious concern regarding public and animal health [2]. It is virtually impossible to contain bacteria, pathogenic or otherwise, within health-care or food animal production systems as there is movement of these organisms along with mobile genetic elements between domestic, domiciliated and wild environments [3,4]. Recent studies reported that antibiotic-resistant bacteria are present in many parts of the globe, including among wild bird species found in remote habitats [5,6]. This presents the possibility of using certain wild animal species as sentinels and potentially a source for the emergence and spread of new AMR profiles. Two such taxa of concern in Ireland are herring gulls (*Larus argentatus*) and deer. Herring gulls are common in certain seaside towns in Ireland and are opportunistic feeders. AMR *E. coli* has been reported in herring gulls previously with the original source being linked to human activity [7]. There has also been a large increase in the range and abundance of deer species in Ireland in recent decades [8] with the increasing likelihood of contact occurring between these animals, food-producing animals and humans. There have been limited studies examining the prevalence of AMR in wild mammals and birds in Ireland. Therefore the aim of this study was to investigate for the prevalence of *E. coli* cultured from the faeces of herring gulls (*Larus argentatus*) and hybrid deer (*Cervus elaphus x Cervus nippon*) in Ireland expressing a multi-drug resistant phenotype.

Samples of herring gull faeces were collected from Howth harbour, a suburb of Dublin City, [coordinates 53°23’22.40 North, 6°04’15.37 West] while deer faeces samples were collected in the Wicklow National Park, Co. Wicklow [coordinates 53°05’50.70 North, 6°15’20.05 West]. Wicklow National Park can be described as upland blanket bog and both of these sites are located on the east coast of Ireland. Thirty individual fresh herring gull and thirty deer faecal samples were collected in June 2012. Sterile swabs were dipped into the recovered faecal samples, swab tips were then broken off into sterile Cary-Blair transport medium, placed into a coolbox and cultured same day upon returning to the laboratory being used to inoculate McConkey agar plates. Plates were incubated for 24 h at 37.5°C to recover *E. coli*. After incubation plates were visually inspected for the presence of typical colonies. Five isolated monoclonal presumptive *E.* coli colonies were picked from each plate and biochemically tested for their indol and citrase reactions. Isolates that tested negative for citrase and positive for indol were confirmed as *E. coli* and PCR was used to determine their phylogenetic group as described below [9]. Confirmed isolates were then re-streaked onto freshly prepared Trypticase Soy Agar (TSA) and these were subsequently used for nitrocefin disc and antimicrobial resistant tests. Antimicrobial susceptibility testing was performed by disc diffusion [10] on Mueller-Hinton agar (MHA) using a panel of 5 antimicrobial agents representing 5 different drug classes, including oxacillin 5 μg, ciprofloxacin 5 μg, rifampicin 5 μg, tetracycline 30 μg and penicillin 5 μg. Antimicrobial agents were chosen to give a range of drug classes including a natural antibiotic, a semi synthetic natural antibiotic and bacteriostatic antimicrobial. Confirmed *E. coli* isolates were inoculated into a 0.85% physiological saline following which the turbidity was adjusted to 0.5-0.7 McFarland. A sterile swab was then used to cover 100% of MHA plate surface. Antimicrobial-containing discs with the drugs indicated above were then placed onto the agar surface and plates were incubated for 24 hours at 37.5°C. Where zones of inhibition were observed, the corresponding diameter was measured (in mm) from 3 different directions using a Vernier callipers and the average of the 3 measurements was recorded [10]. Preparation of DNA and phylogenetic grouping of *E. coli* isolates was determined by PCR [9]. Nitrocefin disc tests were performed on each confirmed *E. coli* isolate that was phenotypically resistant to oxacillin/penicillin to check for β-lactamase production. The development of a red colour on the disc was indicative of the production of a β-lactamase enzyme.

A total of 41 *E. coli* isolates were recovered from the 30 herring gull faecal samples and 51 *E. coli* isolates were recovered from the 30 total deer faecal samples collected, giving 92 isolates confirmed for study. All isolates were susceptible to ciprofloxacin and only one isolate was resistant to tetracycline. In contrast, all of these isolates were resistant to rifampicin, oxacillin and penicillin. Each type of unique resistance profile generate by an *E. coli* isolate was then phylogenetically typed for pathogenicity, where many isolates shared a similar resistance profile only one was phylo-typed. From the herring gull isolates three isolates were phylogenetic group A and seven were phylogenetic group B1. Only one isolate was in the pathogenic phylogenetic group D. A total of six of the herring gull isolates were positive in the nitrocefin test. From the deer isolates, four were phylogenetic group A and seven were phylogenetic group B1 while six isolates were in the pathogenic phylogenetic group D. A total of 12 of the deer isolates were positive in the nitrocefin test (Table 1). Overall the prevalence of resistant isolates was higher in herring gulls with 87% of isolates showing some form of resistance as opposed to deer showing 31%.

**Table 1:** **Summary of genotypic and phenotypic characteristics of AMR *E. coli *** **isolates from wildlife sources**

**Wildlife source**	**Geographic origin**	**No. of *E. coli *** **isolates**	**AMR profile**^**a **^**(%)**					**No. of isolates with nitrocefin test positive**	**Confirmed no. of isolates from each phylo-group**^**b**^			
			**CIP**	**OXA**	**P**	**TET**	**RIF**		**A**	**B1**	**B2**	**D**
Herring gulls	Howth harbour	41	0	100	100	0	100	6	3	7	0	1
Hybrid deer	Wicklow National Park	51	0	100	100	1.9	100	12	4	7	0	6

^a^Antimicrobial compounds are abbreviated as follows: CIP, ciprofloxacin; OXA, oxacillin; P, penicillin; TET, tetracycline; RIF, rifampicin.

^b^Phylo-group: phylogenetic group.

The presence of *E. coli* that are resistant to antimicrobial compounds in wildlife animals such as herring gull and deer species represents a concern to public health with, as yet unquantified effects. To our knowledge, this data represents the first report of bacteria that are resistant to antimicrobial agents among wild mammals in Ireland. One plausible explanation for this could be the interaction of deer herds in the Wicklow area with livestock and farms however, there is no causal evidence as yet. It has been demonstrated that resistant bacteria are present in both Polar Regions confirming that these organisms have the potential to reach geographical extremes and remote environments [5,6]. Nonetheless, an opportunity now exists to use wild-animal species, particularly the birds, as sentinels to monitor the spread of resistant bacteria, since herring gull ecology is well understood. It has been suggested that herring gulls could acquire AMR resistance from human sources and subsequently transfer this resistance to ecosystems previously lacking in resistant bacteria [7,11]. Species in the *Laridae* family migrate long distances and can act as both symptom and source of resistant bacteria over natural protective bio-barriers such as oceans and mountain ranges. There is the additional concern that spill over of these bacteria of importance to human and animal health, into wild species could result in wildlife hosts functioning as a reservoirs and vectors for reintroduction to the human populations or introduction to farm animals. Future research focused on the role of wild birds and mammals, as a sentinel species contributing to the dissemination of antimicrobial-resistant bacteria is important. An understanding of the selection pressures, evolution and dissemination of resistance genes in pathogenic organisms is not complete without fully understanding the role of wild-species and natural environments in the process [12]. AMR has been subject to substantial research efforts however, it still remains a massive global public health challenge. Effective antimicrobial therapy using the available arsenal of drugs is important to protect animal and human in public health into the future. Thus a complete understanding of the role played by antimicrobial-resistant bacteria in the domestic, domicilicated and wild environments is required to provide scientifically-sound mitigation measures capable of addressing this public health challenge.

## Competing interests

The authors declare that they have no competing interests.

## Authors’ contributions

SS performed the sampling, analysis and wrote the draft of this report. SF and BJMcM supervised all stages of the work presented in this report and critically read the report. JW assisted in the analysis. All authors read and approved the final manuscript.
